# Even stressed cells are individuals: second messengers of free radicals in pathophysiology of cancer

**DOI:** 10.3325/cmj.2012.53.304

**Published:** 2012-08

**Authors:** Morana Jaganjac, Tamara Čačev, Ana Čipak, Sanja Kapitanović, Koraljka Gall Trošelj, Neven Zarković

**Affiliations:** Division of Molecular Medicine, Ruđer Bošković Institute, Zagreb, Croatia zarkovic@irb.hr

## Abstract

**Abstract:**

Pathophysiological processes associated with disturbances in cell and tissue oxidative homeostasis, are associated with self-catalyzed process of lipid peroxidation. The end products of lipid peroxidation are reactive aldehydes such as 4-hydroxy-2-nonenal (HNE), acting as “second messengers of free radicals.” Although reactive aldehydes were first recognized only as cytotoxic, new evidence has come to light, related to their cell growth regulatory functions achieved through cell signaling. The variable appearance of HNE in several organs indicates that its mode of action might be related to an individual cell stress adaptation. The underlying mechanism could be that specific mutations and epigenetic changes on one hand interfere with hormesis on the other. The precise role of oxidative stress and lipid peroxidation in these processes still needs more clarification at molecular level. Finally, an individual approach to each patient, based on the individual cell response to stress, opens a new possibility of integrative medicine in cancer treatment and strongly supports modern concepts of personalized medicine.

Our everyday exposure to physical exercise and psychological (eg, stress at work) or environmental (eg, pollution, cigarette smoke) stress provoke changes on the cellular level resulting in excessive production of reactive oxygen species (ROS). In addition, ROS are also continuously formed endogenously in small amounts during the normal cell metabolism (eg, electron transport chain in mitochondrion). ROS are ubiquitous, short-lived chemically-reactive molecules containing oxygen that can react with surrounding molecules at the site of formation. Therefore, cells have evolved efficient enzymatic and non-enzymatic antioxidant defense mechanisms to deal with ROS. The condition under which antioxidant mechanisms are overwhelmed by ROS is known as oxidative stress. Mild oxidative stress occurs on a daily basis and is a key factor in maintaining homeostasis. However, strong, acute, or chronic oxidative stress occurring as a consequence of various harmful conditions (ie, long-lasting infections, or autoimmune diseases) disrupts this delicate homeostasis. The inability to maintain homeostasis and deregulated molecular, cellular, and physiological pathways, which are activated to repair stress-induced damage, results in increased vulnerability, age- and stress-associated disorders and the onset of diseases like metabolic syndrome and malignant diseases (Figure 1). Due to scope and space limitations, this text will focus on the role of oxidative stress only in carcinogenesis.

**Figure 1:**
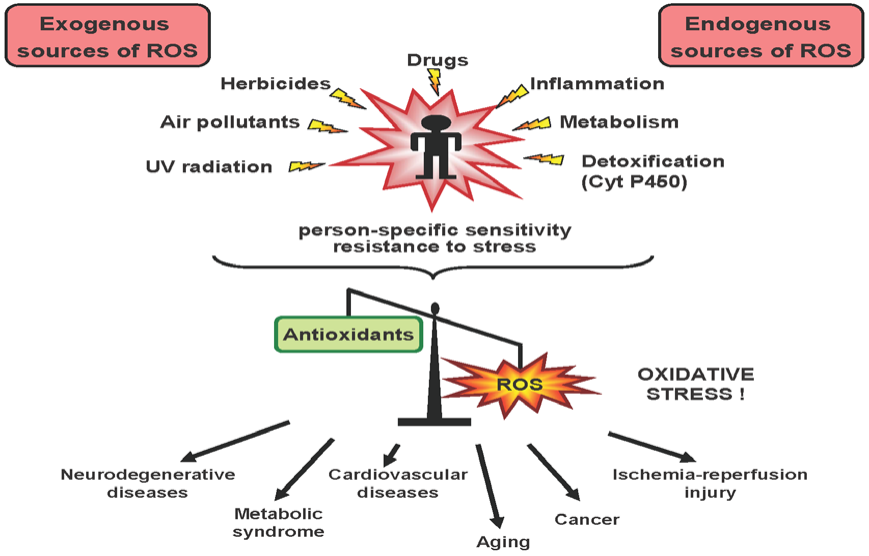
Disruption of homeostasis in human body leads to development of various oxidative stress-associated diseases in a person-dependent manner.

## The effects of oxidative stress on macromolecules

At low physiological levels, ROS are indispensable in numerous biochemical processes, functioning as redox messengers and important molecules in intracellular signaling. In addition to having an important role in cellular differentiation, proliferation, and apoptosis, ROS are also key players in inflammatory processes and defense against microorganisms. However, at high levels, ROS may oxidize the DNA molecule, proteins, lipids, and carbohydrates, mediating numerous redox-mediated pathological conditions. This is mostly due to the rapid reactions of free radicals, which have unpaired electrons reacting aggressively with the nearby macromolecules.

The oxidation of proteins causes the formation of carbonyl groups, mainly on Pro, Arg, Lys, and Thr amino acid side chains. The oxidative cleavage of proteins, either by the α-amidation pathway or by the oxidation of glutamyl side chains also results in generating protein carbonyl derivates (1). A common effect of protein carbonylation is the loss or gain of function of target proteins related to age-related conditions and various diseases (2). Modifications of metabolic and structural proteins cause protein dysfunction, altering protein processing and trafficking, generating tissue damage and, consequently, being an underlying pathogenic mechanism for numerous human diseases (3).

ROS can oxidatively damage DNA, resulting in oxidized nucleic acid bases, apurinic/apyrimidinic DNA sites, and single-strand or double-strand DNA breaks (4). Hydroxyl radical (OH) reacts with heterocyclic DNA bases by addition and abstraction, yielding reactive purine OH-adduct radicals, pyrimidine OH-adduct radicals, and the allyl radical. Further reactions of these radicals may result in a variety of final products (5). The guanine oxidative product, 8-oxo-2’-deoxyguanosine (8-oxodG), is one of the best studied oxidative lesions and is widely used as a biomarker for oxidative stress and carcinogenesis. Cancer is nowadays considered as persistent oxidative stress disorder (6).

The mutagenic potential of 8-oxodG is reflected in its miscoding properties. Although it can form Watson-Crick base pairs with 2-deoxycytidine, it also readily mispairs and forms Hoogstein base pairs with 2-deoxyadenosine during replication leading to GC→TA mutations (7). These transversions were observed in numerous cancers, such as skin and lung cancer, in the *ras* oncogene and *p53* tumor suppressor gene (8,9). Much of the research examining the consequences of oxidative damage to DNA focused on mutations. However, emerging work clearly showed an extensive presence of epigenetic changes. When adjacent to 5′-methyl-cytosine (5-mC), 8-oxodG diminishes the binding of methyl binding proteins, MBP, as clearly shown for MeCP2 (10). On the other hand, the sensitivity of the methyl group of 5′-mC to oxidation, leading to generation of 5′-hydroxymethylcytosine (5-hmC), was known for a long time (11). What was not known at the time of this discovery is that the formation of 5-hmC interferes with many events in chromatin condensation cascade, resulting in potentially heritable epigenetic alterations. The research on the activity of CMV promoter in HeLa cells extracts, showed that the presence of 5-hmCs in the gene promoter inhibited transcription, while their presence in the gene body did not inhibit transcription directly (12).

Other targets of ROS are unsaturated fatty acyl groups in membranes or storage lipids. Their ROS-induced peroxidation and breakdown results in the destruction of biomembranes. The final products of lipid peroxidation are reactive aldehydes such as 4-hydroxyalkenals and other similar α,β-unsaturated aldehydes, among which, of particular biochemical and biomedical relevance, are 4-hydroxynonenal (HNE), malondialdehyde (MDA), and acrolein (13). A large number of these aldehydes were isolated from biological samples, where they may promote and reinforce cell damage induced by oxidative stress. Lipid-derived aldehydes are more stable than ROS and they can diffuse across membranes and reach targets distant from the initial site of oxidative injury (2). Such reactive aldehydes are subject to Michael addition reactions, with the nucleophilic side chains of lysine, histidine, and cysteine residues resulting in protein carbonylation (14,15). The aldehydes are very effective in binding to DNA, leading to adduct formation and eliciting mutagenic effects (13). Michael addition of α,β-unsaturated aldehydes to deoxyguanosine yields N(2)-(3-oxopropyl)-dG adducts. Reversible cyclization of N1 with the aldehyde yields 1,N(2)-dG adducts, which can interfere with replication and transcription of DNA (16,17). It was found that HNE causes G→T transversions in the p53 tumor suppressor gene at codon 249 (18). Acrolein-derived 1,N(2)- γ-hydroxypropano deoxyguanosine (γ-HOPdG) blocks mammalian pol δ and pol ϵ DNA polymerases (19). HNE, which triggers apoptosis through c-Jun N-terminal kinase (JNK) activation, interacts and increases induction of remaining TGF-β1 pathways in tumor cells. Therefore, they become resistant to TGF-β1-mediated growth inhibition, thus contributing to inhibition of the tumor growth (20). Additionally, HNE plays a role in modulation of cell growth, differentiation, cell signaling and apoptosis (21,22). This small and highly reactive molecule, considered to be a second messenger of free radicals and major bioactive marker of oxidative stress, acts as a strongly reactive molecular link between genome and proteome through epigenome (23).

## Involvement of oxidative stress in carcinogenesis

**Figure 2:**
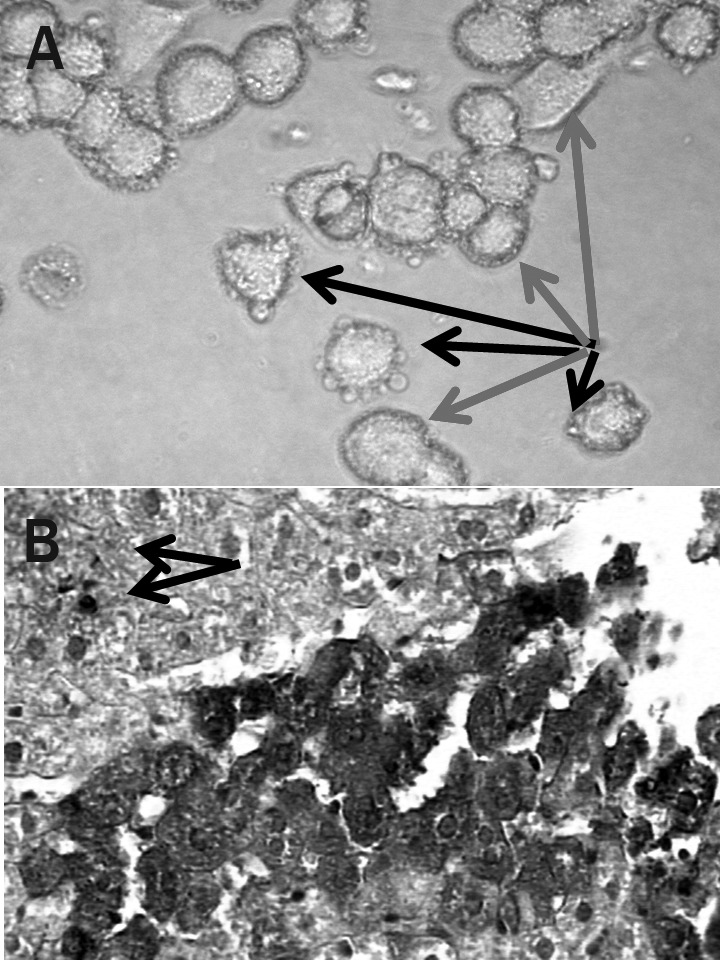
Microphotographs revealing individuality of the cellular 4-hydroxy-2-nonenal (HNE). Although standardized line HeLa cells (**A**) are considered all to be identical, a treatment with 50 μM HNE for 30 minutes revealed great differences in their individual reactivity to the toxic dose of the aldehyde. Thus, while some cells showed membrane lipid peroxidation damage (“blebs” indicated by black arrows), their neighbor cells did not show any signs of the damage (gray arrows). Similarly, 30 minutes after biopsy needle puncture the affected liver cells (**B**) showed strong immunopositivity for HNE (darker shade), while their neighbor cells were negative for the aldehyde (lighter shade). However, some cells, even remote, began generating HNE indicating individual sensitivity of the cells to the damage signaling (indicated by the black arrows).

ROS have a dual role in tumor biology (24). The manner in which they act depends on both the specific location where they are produced and the number of individual ROS. Previously, we have reported the involvement of oxidative stress in both tumor progression (25,26) and regression (27-29), describing its effect in different malignancies, namely melanoma B16F10, Ehrlich ascytic tumor, and Walker 256 carcinoma (W256). It is well known that both genotoxic and non-genotoxic mechanisms modulate gene activity, which occurs – at the level of the epigenome, before any detectable structural genetic change (ie, DNA mutations). Oxidative stress is involved in both types of mechanisms and it has a very important role in the process of carcinogenesis (30). Accordingly, rather than structural molecular changes in a specific gene or gene cluster, there are “hallmarks of cancer” that enable a cell to change its transcription machinery, become malignant, and metastasize. The reason for these early changes is closely related to aberrant DNA methylation, which is considered to be among the earliest changes to occur in carcinogenesis. Aberrant DNA methylation is extensively present in cancer cells as global hypomethylation and focal, specific hypermethylation in promoters of tumor suppressor genes, leading to their silencing. For example, *GSTP*1 codes for a detoxifying enzyme that catalyzes conjugation reactions between potentially damaging oxidants. This gene is the most frequently methylated, and, consequentially, silenced, in more than 90% of prostate cancer lesions, approximately 70% of high grade neoplasia (PIN) lesions, but very rarely in normal prostate and/or benign prostatic hyperplasia (PHB) (31). These methylation aberrations, resulting in entirely changed gene activity, inevitably lead to key capabilities that have been summarized in an anthological review by Hanahan and Weinberg (32) as sustained proliferative signaling, evasion of growth suppression, resistance to cell death, replicative immortality, induction of angiogenesis, and activation of invasion and metastasis. A recent addition to these six core capabilities are the deregulation of cellular energetics and metabolism as well as the evasion of the immune destruction (33). Oxidative mechanisms possess a potential role at all stages of carcinogenesis. In addition to cancer cells, tumors interact with a repertoire of normal cells, contributing to the acquisition of hallmark traits by creating a “tumor microenvironment” (34). We found that normal cells in the vicinity of malignant ones are in particular stressed by HNE or acrolein, mostly likely due to the cancer progression and surrounding tissue destruction (35,36). On the other hand, cancer cells have very dynamic turnover of lipids, thus avoiding cytotoxic effects of lipid peroxidation and growth regulating effects of the reactive aldehydes. This is especially important for cancer progression because malignant cells often have reduced antioxidant capacities, if compared with the non-malignant counterpart cells, which makes them more susceptible to the toxicity of the aldehydes (37,38). It is also important to note that for both malignant and non-malignant cells, the oxidative homeostasis is cell-specific, showing extreme variation in the cellular HNE levels both in vitro and in vivo and causing differential sensitivity of apparently identical cells to oxidative stress (Figure 2). Eventually, (dis)balance of oxidative homeostasis in malignant and surrounding nonmalignant cells might be crucial in determining the growth characteristics of the cells and the spread of oxidative damage mediated by reactive aldehydes (39,40).

## Personalized cancer medicine

It is now evident that, although general mechanisms of tumor development and progression can be summarized by the acquisition of postulated hallmark capabilities, the actual processes taking place in each specific tumor type or subtype, or even in some of the tumor cells, are unique and specific for each individual patient as well as for each cell. It is true that tumor cells, in order to progress, need to overcome common obstacles in their way. The way they do it is through a distinctive set of epi-mutations, structural mutations, and various types of adaptations, eg, in a state of oxidative stress. Today, we know that different parts of tumor exhibit specific cell subpopulations, harboring differently methylated parts of DNA as well as different mutations, possibly serving as driver mutations that may become useful for tumors to overcome the effects of a specific targeted therapy. Another level of complexity is added by the specific characteristics of stromal cells, producing high amounts of TGFβ, which promote epithelial-mesenchymal transition (EMT) during tumorigenesis (41). The EMT phenomenon is characterized by the remodeling of epithelial cell-cell and cell-matrix adhesion contacts, repression of epithelial markers, induction of mesenchymal markers, and the acquisition of motile capacity, leading to an invasive form of cancer. This reversible process is closely related to oxidative stress, as very recently shown in an experimental model of lung cells exposed to nickel. The exposure induced very strong oxidative stress (measured through generation of ROS) and ROS-mediated EMT. The underlying molecular mechanism was, at least partially, hypermethylation of E-cadherin promoter, with its consequential silencing (42).

## Conclusions

**Figure 3:**
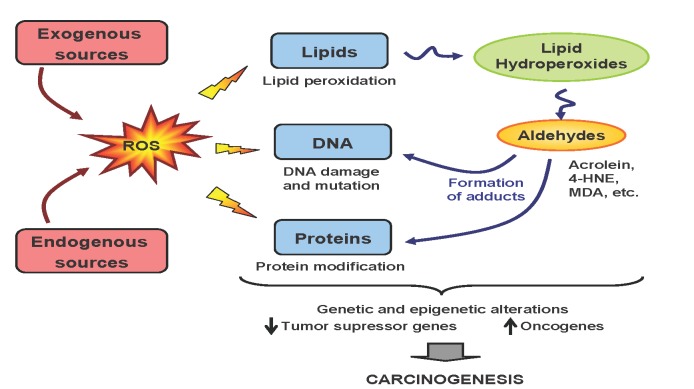
Potential mechanisms of oxidative stress induced genetic and epigenetic alterations leading to carcinogenesis.

Oxidative stress and lipid peroxidation are present in human body cells throughout life, playing an extremely important role in survival. Depending on the severity of oxidative stress and lipid peroxidation, the effects on cells and the organism vary from positive (hormesis) to noxious, which lead to the occurrence of various diseases such as cancer (Figure 3). Persistent oxidative stress and excess lipid peroxidation cause DNA damage that, along with deregulation of cell homeostasis, lead to carcinogenesis. Lipid peroxidation acts as a double-edged sword in carcinogenesis, exhibiting either a pro- or anti-cancerous effect. Lipid-derived reactive aldehydes, in particular HNE, act as second messengers of free radicals and as growth-regulating factors, hence they are of high importance in determining oxidative homeostasis on cellular level and on the level of the organism. Monitoring the level of oxidative stress should be an obligatory component of personalized medicine in particular in oncology. to recognize specific molecular changes present in a specific tumor (or in a subset of tumor cells) of a specific patient at the time of the diagnosis, or during therapy. This individualized approach, based on repeated genetic profiling of a given tumor, therapy monitoring, and the possible emergence of acquired resistance (43), joined with relevant information on the methylome, transcriptome, proteome, and on both oxidative stress and lipid peroxidation levels, could significantly contribute to an improvement of therapy and prognosis in cancer patients. Rapid technological expansion has generated valuable tools for analyzing the tumor “global picture.” While high-throughput sequencing and microarray techniques might prove helpful in identifying possible targets for anticancer therapy, epigenomic and proteomic analysis, including aldehyde-adducts, may be used for precise identification of specific tumor biomarkers as well as markers of response to therapy (44), especially in oxidative stress-related malignancies.
